# The potential role of Artificial Intelligence in transforming childhood cancer care

**DOI:** 10.1016/j.eclinm.2024.102705

**Published:** 2024-06-20

**Authors:** eClinicalMedicine

Childhood cancer represents an area of major within health care, marked by its profound impact on young lives and the multitude of challenges it poses in diagnosis and treatment. Every year, approximately 300 000 children aged 0–19 years are diagnosed with cancer worldwide. Unlike adult cancers, which are thought to be caused by a combination of age-related, environmental, and genetic factors, childhood cancers are predominantly driven by genetic abnormalities, requiring prompt and precise intervention. 5-year survival rates can range from almost zero for cancers such as diffuse intrinsic pontine glioma (2%)a type of brain cancer, to as high as 90% for the most common types of childhood cancer such as acute lymphoma leukaemia, Hodgkin lymphoma, and Wilms tumours. Despite progress, several challenges persist in diagnosing and treating childhood cancers. Early detection is often difficult, due to the non-specific nature of initial symptoms, and treatment regimens can be highly intensive and toxic, posing substantial risks to young patients whose bodies are still developing. Therefore, there is an urgent need for improvements in childhood cancer diagnosis, treatment, and care.

Artificial Intelligence (AI) has achieved noteworthy successes in adult oncology, including enhanced diagnostic accuracy through advanced imaging analysis, personalised treatment plans via genomic data interpretation, and improved patient outcomes through predictive analytics. These developments have improved the earlier diagnosis, enhanced the effectiveness of treatments, and helped the management of numerous adult cancers. Therefore, AI has enormous potential for redefining health care for both adult and childhood cancers. However, its applications in paediatric oncology have lagged due to limitations, such as the rarity and diversity of childhood cancers, scarce paediatric-specific data, and ethical concerns related to young patients—where it remains largely unexplored.

One of the major barriers is the rarity of paediatric malignancies, which account for less than 1% of all cancer diagnoses annually in the USA. This rarity inherently complicates efforts to collect sufficient data for useful analysis and model development. Additionally, with their diversity and distinct biological characteristics, paediatric cancers have another layer of complexity to their development and treatment protocols as well as outcomes. Consequently, AI models must be adaptable and versatile enough to accommodate this diversity.

The ethical considerations surrounding paediatric cancer research present unique barriers. Children are considered a vulnerable population, and stringent regulations govern their participation in research studies. Obtaining informed consent from parents or guardians, as well as assent from older children and the ethical committee, is crucial, but it can be logistically and emotionally challenging. Additionally, ensuring the privacy and confidentiality of paediatric patients’ medical data is paramount, demanding robust data security measures. Furthermore, the limited availability and accessibility of comprehensive paediatric cancer datasets hinder progress in AI applications. Data fragmentation and siloed information systems are common hurdles that prevent the collection and standardisation of data required for AI model training and validation.

At the European Society for Paediatric Oncology (SIOPe) Annual Meeting in Milan 2024, Dr Norbert Graf from the Saarland University presented the UNICA4EU, a pioneering project focused on the intersection of AI and paediatric oncology, which serves as an example of AI implementation in childhood cancers. Its primary objective is to leverage AI technologies to improve the diagnosis, treatment, and overall care of children with cancer. Recognising the unique challenges posed by the rarity and diversity of childhood cancers, UNICA4EU aims to create innovative solutions that can enhance clinical outcomes and streamline health-care delivery in Europe. The project aggregates and analyses data from various sources, including clinical records, genomic studies, and medical imaging and applies advanced AI algorithms to analyse diagnostic data, such as MRI scans and biopsy results, to detect cancer at its earliest stages. Additionally, AI tools are used to predict patient prognosis, helping clinicians make informed decisions about treatment plans. This includes selecting the most effective chemotherapy drugs, identifying potential side-effects, and adjusting treatment plans based on real-time patient responses. By creating a platform for sharing data, resources, and expertise, the project intends to accelerate the development of AI applications and ensure that the latest innovations are accessible and quickly translated into clinical practice. Furthermore, the project offers resources and training programmes to help clinicians and researchers understand and effectively use AI tools in their practice, ensuring that the benefits of AI are fully realised in paediatric oncology. Programmes such as the UNICA4EU project could contribute to improving early identification and lowering potential risks associated with prolonged and toxic therapies, resulting in better outcomes for younger patients with cancer.

AI-driven solutions have shown promise in expediting research, facilitating data-driven decision making, and equipping health-care providers with advanced tools and insights. However, unlocking the full potential of AI in childhood cancer demands collaborative efforts from health-care professionals, researchers, policy makers, and technology developers. The potential impact of AI on childhood cancer offers a pivotal opportunity to transform the approach to diagnosis, treatment, and care for young patients. Overcoming the barriers and increasing the implementation of AI in paediatric cancer is essential for enhancing outcomes and ultimately saving children’s lives.

